# Nomogram model to predict postoperative infection after mandibular osteoradionecrosis surgery

**DOI:** 10.1038/s41598-017-03672-2

**Published:** 2017-06-14

**Authors:** Zhonglong Liu, Tianguo Dai, Zhonghe Wang, Zhiyuan Zhang, Weiliu Qiu, Yue He

**Affiliations:** 1 Department of Oral Maxillofacial & Head and Neck Oncology, Shanghai Ninth People’s Hospital Affiliated to Shanghai Jiao Tong University School of Medicine, Shanghai, 200011 China; 2 Department of Stomatology, Panzhihua Central Hospital of Sichuan Province, No. 34, Panzhihua Street Middle Section, Panzhihua City, 617067 China; 3 Department of Radiotherapy, Shanghai Ninth People’s Hospital Affiliated to Shanghai Jiao Tong University School of Medicine, Shanghai, 200011 China

## Abstract

Osteoradionecrosis of the mandible (ORNM) is one of the most dreaded complications of radiotherapy. The poor healing capacity of soft tissue after radiation may lead to surgical failure. The current study was designed to identify prognostic factors for postoperative infection (PPI) and propose corresponding prophylaxis and intervention protocols. A retrospective study was conducted concerning ORNM patients from 2000 to 2015. A risk-stratification score and nomogram model were established to predict the risk of PPI. A total of 257 patients were analyzed, and the total incidence of PPI was 23.3% (60/257). In multiple logistic regression analysis, radiation dose $\geqslant$80 Gy (versus <80 Gy, OR = 2.044, P = 0.035, 95% CI: 1.05–3.979), bilateral ORNM (versus unilateral, OR = 4.120, P = 0.006, 95% CI: 1.501–11.307), skin fistula (versus none, OR = 3.078, P = 0.040, 95% CI: 1.05–9.023), and implant utilization (versus none, OR = 2.115, P = 0.020, 95% CI: 1.125–3.976) were significantly associated with PPI. The susceptibility to PPI in patients with risk-stratification scores of 14–22 was 2.833 times that of patients with scores of 7–13, and 7.585 times that of cases defined as scores of 0–6. The discrimination capability of the nomogram model was estimated using a ROC curve with an AUC of 0.708, revealing potentially useful predictive abilities. In conclusion, current risk-stratification scores and nomogram models effectively predicted the risk of PPI in ORNM patients.

## Introduction

Radiotherapy is common among the multidisciplinary therapeutics used to treat head and neck cancers. Osteoradionecrosis of the mandible (ORNM) is a serious, radiation-induced complication characterized by sequestra or devitalized bone formations with soft tissue destruction^1, 2^. Without medical intervention, this disease can develop into skin fistulae, bone exposures, mouth opening limitations, and pathological fractures, imposing tremendous psychological and physical trauma on patients. Therapeutic methods, including conservative treatment, sequestrectomy, segmental resection and hemimandibulectomy with simultaneous reconstruction, are selected based on the severity of ORNM^3–5^. Because of radiation damage to soft tissue (fibrosis and scar) and microcirculation, ORNM patients experience a higher risk of postoperative morbidities, such as delayed wound healing, dehiscence, swelling, fistula and infection, relative to non-pre-radiated surgeries^6, 7^. Several factors, including mucositis, decreased salivary secretion, mucosal fibrosis and poor oral hygiene, may lead to wound infection and subsequent orocutaneous fistula; these are formidable complications for surgeons to address during the perioperative period^7^. Once an infected fistula evolves into titanium plate extrusion or even bone exposure, plate removal or bone resection combined with vascularized flap coverage should be performed to control this relapse^8^. Furthermore, postoperative infection (PPI) may facilitate disease progression in patients who have undergone surgery for ORNM, even if a radical resection was performed. Considering these challenges, we set postoperative infection of ORNM as the primary outcome; the objectives of the study are to establish a predictive model for susceptibility to PPI and to propose precautionary protocols. To our knowledge, our study has the largest sample size of all studies examining prognostic parameters for PPI of ORNM. Based on this study, we hope to provide meaningful recommendations for the treatment of ORNM.

## Methods

### Study design and sample

To address this clinical issue, we designed and implemented a retrospective study. We used the files of ORNM patients who received treatment at the Department of Oral Maxillofacial & Head and Neck Oncology between 2000 and 2015. We only conducted our study on patients who met the following criteria: (1) patients diagnosed with ORNM with no evidence of primary tumor recurrence; (2) patients who received surgical treatment, including sequester, sequestrectomy, segmental or partial resection, hemimandibulevtomy; (3) patients who received treatments in single institution; (4) patients who did not die during perioperative period (beginning at the time of hospital admission and ending at patient discharge); and (5) patients with complete treatment records and follow-up information. We confirmed that all methods were carried out in accordance with study guidelines and regulations; all experimental protocols were approved by the Ethic Committee of Shanghai 9th People’s Hospital, and informed consent was obtained from all subjects.

### Study variables and estimation model

The demographic and comorbidity features included age at surgical treatment, gender, smoking, alcohol consumption and systemic disease; ORNM-related characteristics before surgery included primary tumor, mandibular surgery prior to radiotherapy, radiotherapy (the dose and the time interval between radiation completion and the onset of ORNM, which was counted by month), tooth extraction and hyperbaric oxygen therapy; and ORNM clinical manifestations included morphological changes, spontaneous pain, numbness, mucosal defects, skin fistulae, orocutaneous fistulae, bone exposure, titanium plate exposure, purulent discharge, limitation of mouth opening (categorized as mild, moderate, severe or trismus) and pathological fracture. Particular emphasis was placed on ORNM classification and stage^1^. Data regarding treatment consisted of different surgical therapies and titanium plate utilization. All aforementioned variables were defined as primary predictive factors, and the outcome variable was postoperative infection. Follow-up began at the time-point when patients finished surgical treatment to three months after surgery. An association study of primary predictive factors with the outcome variable was performed to filter the factors with predictive roles; these factors were then recombined to establish a risk-stratification score and nomogram model to predict the PPI of ORNM.

### Statistical analysis

Sociodemographic and clinical variables were analyzed using descriptive statistics. The association between primary predictive factors and PPI was tested in a univariate analysis (Chi-square tests, *P* < 0.05 was deemed statistically significant). Those factors with *P* < 0.05 were then entered into multiple logistic regression analysis to eliminate the reciprocal influence and determine the final predictive role. *P* value, odds ratio and 95% confidence intervals were recorded. For the risk-stratification score, the score was assigned to predictors filtered from multivariate analysis according to the regression coefficient obtained from the logistic model. This score was subsequently analyzed in Chi-square tests to clarify its predictive role of PPI. For better analysis, we also established a nomogram model to show the odds ratio of patients with different risks and scores. We further assessed the model’s performance via discrimination (ROC curve, area under curve, AUC), and model fitness was tested using bias-corrected calibration curve with 1000-sample bootstrapping for the prediction of PPI. Bootstrapping was used as internal validation. Statistical analyses were performed with SPSS software (version 21.0; SPSS, Chicago, IL) and R version of 3.3.1 (http://www.rproject.org).

## Results

### Clinical characteristics of study cohort

**Figure 1:**
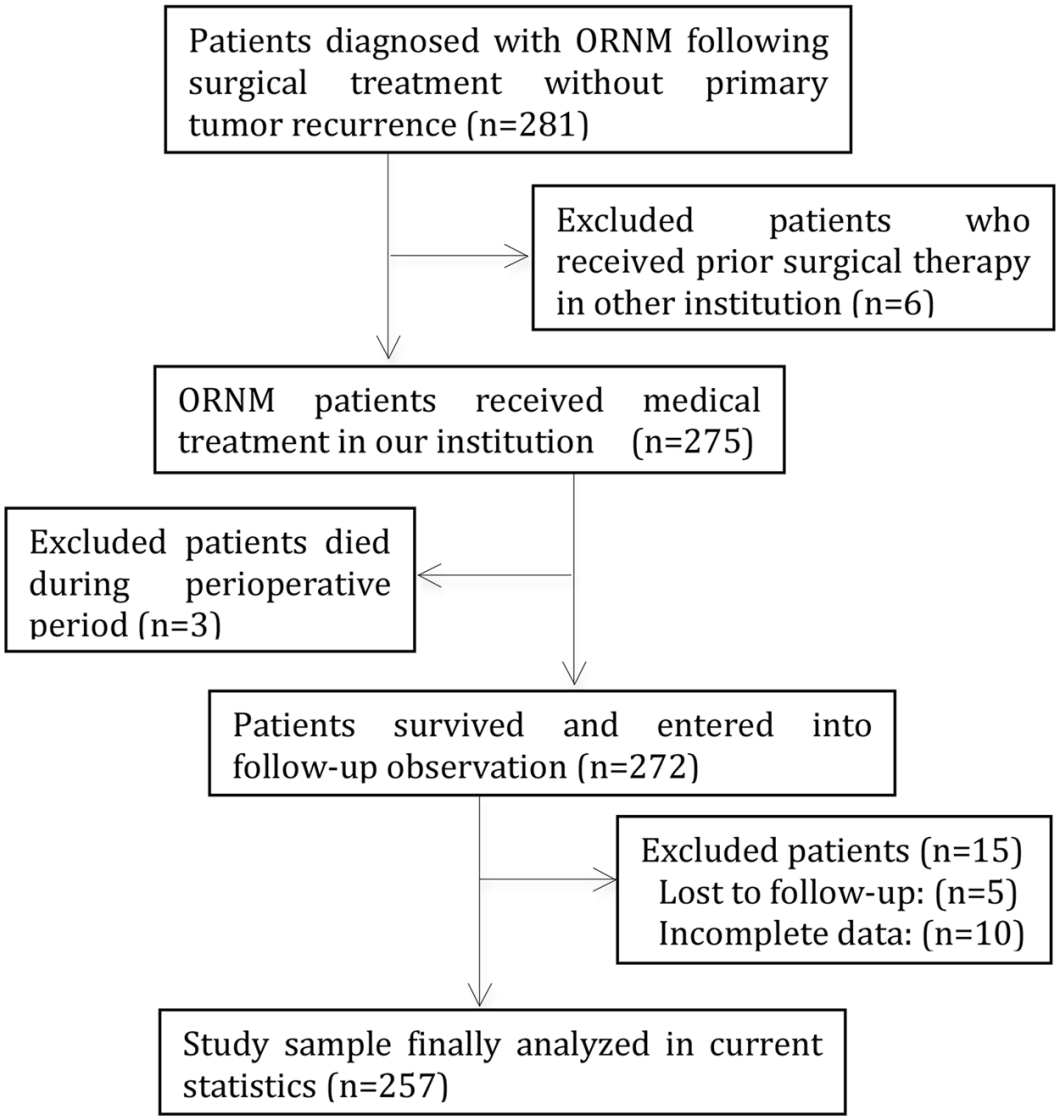
Flow diagram of patients selection.

Twenty-four patients were excluded according to the inclusion criteria, and a total of 257 patients were analyzed in the study (Fig. 1). Three patients died during the hospital-stay period: one as the result of an electrolyte disturbance, one as the result of a carotid hemorrhage, and one as the result of heart disease. Thus, we failed to determine the PPI information within the first three months after surgical treatment for their deaths during the hospital-stay period. The age distribution ranged from 24 to 92 years (mean: 55.7, median: 55), with 170 (66.1%) patients younger than 60 years old. The group was mostly composed of male patients (n = 202, 78.6%). Other demographical characteristics are summarized in Table 1.

**Table 1:** Baseline characteristics of ORNM patients.

	No. of Patient (N%)
Age (Yr)	
<60	170 (66.1)
$\geqslant$60	87 (33.9)
Gender	
Male	202 (78.6)
Female	55 (21.4)
Smoking	
None	174 (67.7)
Yes	83 (32.3)
Alcohol consumption	
None	198 (77.0)
Yes	59 (23.0)
Systemic disease	
None	199 (77.4)
Yes	58 (22.6)

**Figure 2:**
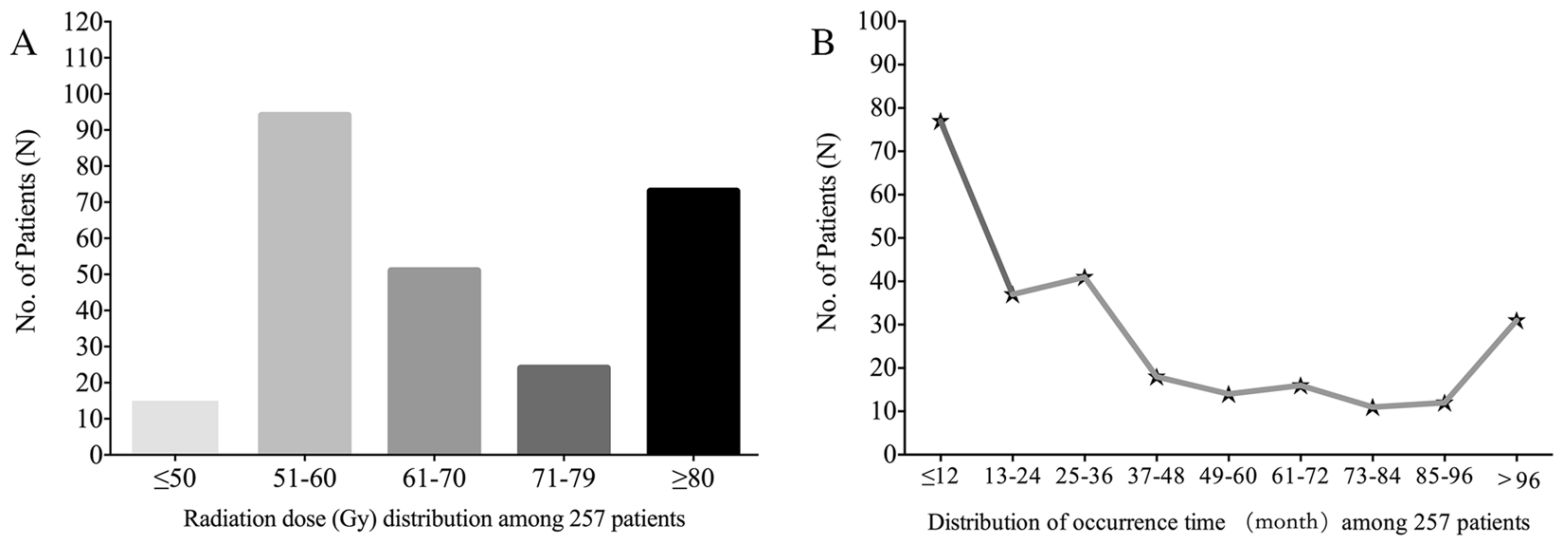
(**A**) Radiation dose distribution among 257 patients with 73 cases received dosage $\geqslant$80 Gy; (**B**) Distribution of patients among varied time interval between radiotherapy complete and the onset of ORNM.

Among 257 cases, the most common primary tumor was nasopharyngeal carcinoma (n = 96, 37.4%), followed by tongue cancer (n = 49, 19.1%) and buccal malignancy (n = 18, 7.0%) (Table 2). Among patients receiving extended resections of malignant neoplasms, 54 cases (21.0%) underwent simultaneous marginal resections of the mandible, and an additional 11 patients (4.3%) received segmental resections. Subsequent X-ray radiation, ranging from 35 to 144 Gy (mean: 75.35; median: 70), was performed in all patients (Fig. 2A).

**Table 2:** Primary tumor before radiation.

Primary location	No. of cases	%
Nasopharyngeal	96	37.4
Tongue	49	19.1
Buccal	18	7.0
Mouth floor	17	6.6
Gingiva	16	6.2
Oropharyngeal	13	5.1
Jaw	9	3.5
Tonsil	8	3.1
Palate	7	2.7
Parotid gland	7	2.7
Submandibular gland	7	2.7
Neck	6	2.3
Others	4	1.6

### Clinical presentations and staging of ORNM

**Figure 3:**
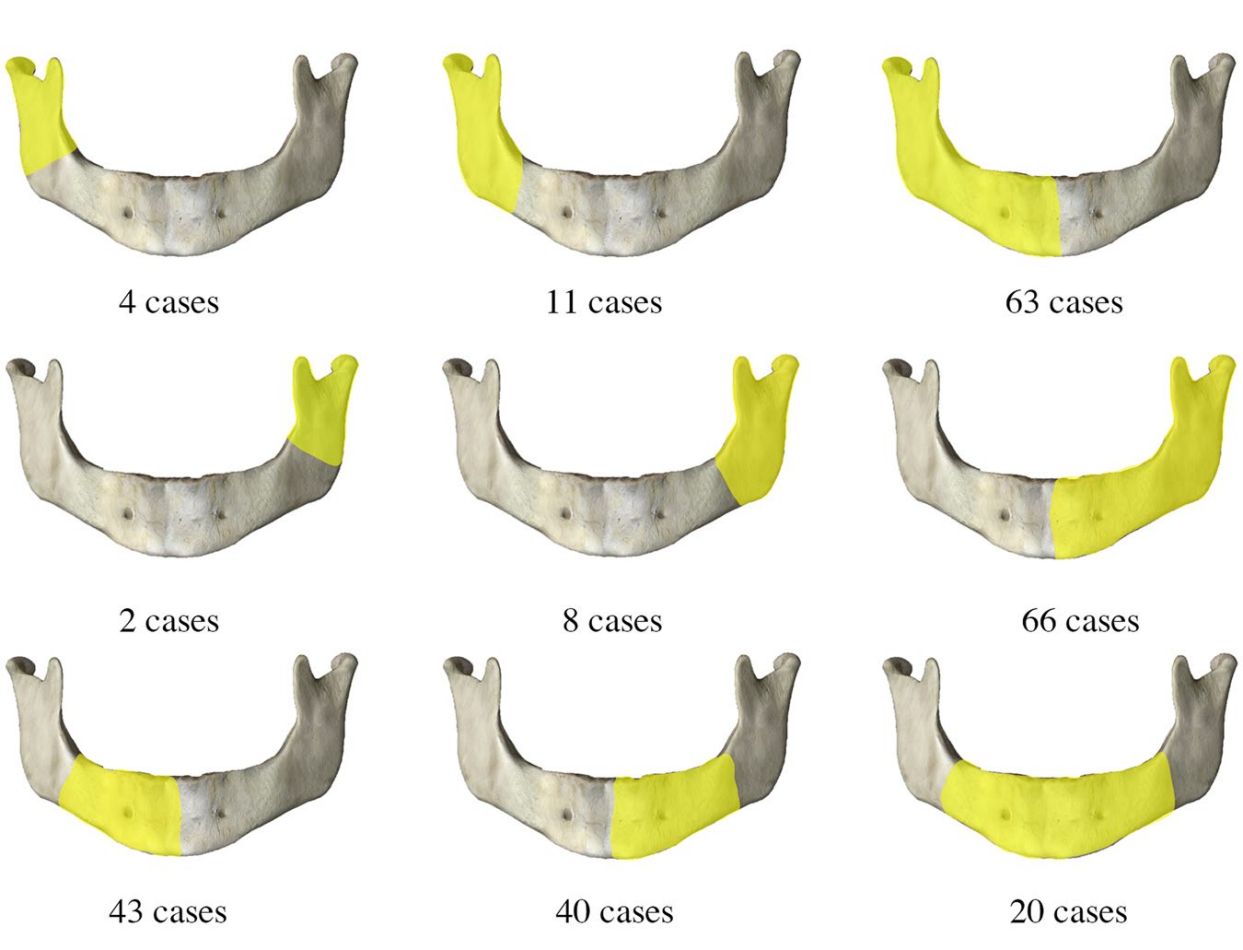
Distribution of lesion sites of ORNM.

The time interval between radiotherapy completion and the onset of ORNM varied from 1 month to 456 months (mean: 46.9; median: 36); cases diagnosed within 36 months accounted for 60.3% (n = 155) (Fig. 2B). With regards to the lesion site of the mandible, most cases (n = 237, 92.2%) were found unilaterally in the mandible (116 in left and 121 in right), and the remaining 20 patients were diagnosed with bilateral ORNM. From anatomic analysis, bone destruction mostly occurred in the mandibular body combined with the angle and the ramus (n = 129, 50.2%) (Fig. 3).

**Figure 4:**
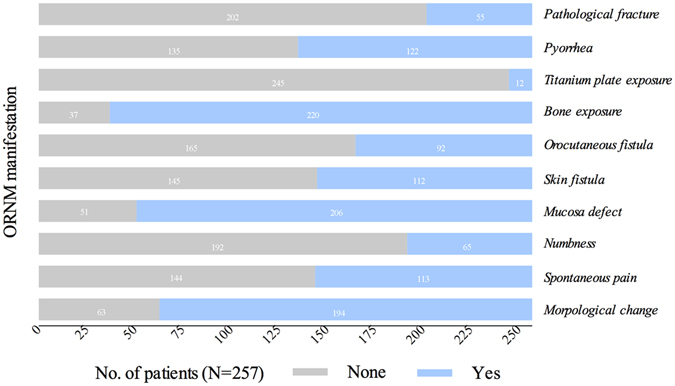
Clinical manifestations of ORNM and their distribution among patients.

ORNM in its early stage is easily overlooked because it is symptomless and shows little change in both radiographic and clinical examinations. The patients sought outpatient service after experiencing obvious symptoms or symptoms beyond their tolerance, such as morphological changes, spontaneous pain, numbness, mucosal defects, skin fistulae, orocutaneous fistulae, bone or titanium plate exposure, pyorrhea, and pathological fracture. Detailed information on this topic is provided in Fig. 4. The majority of patients (n = 240, 93.4%) complained of limitations in mouth opening; this limitation was mild in 19 patients, moderate in 74 patients, severe in 140 patients, and trismus in 7 patients. All patients were categorized into variable stages per the staging system mentioned above. Most of the cases were classified as Stage II (178/257, 69.3%), followed by Stage III (55/257, 21.4%) and Stage I (24/257, 9.3%).

### Surgical treatments and postoperative infection

Surgery was performed on all cases in our study, and the resection margin adhered to the criteria for the appearance of well-vascularized and infection-free bone. Twenty-nine patients (11.3%, Grade I = 24, Grade II = 5) were treated with curettage/sequestrectomy and primary closure of the wound. The remaining 228 patients (88.7%; Grade II = 171, Grade III = 55) received extended resection; of these, 156 patients (68.4%) underwent simultaneous flap tissue transfer: 71 patients from the pectoralis major myocutaneous flap, 63 from the fibular flap and 11 from the anterolateral thigh flap. Titanium plates were used in 106 patients (41.2%) to maintain the anatomic arch of the mandible after segmental resection or to fix the binding interface between the remaining mandible and bone graft (Table 3).

**Table 3:** Therapeutic methods and implant utilization.

Methods	Total (%)
Curettage/Sequestrectomy	29 (11.3)
Extensive resection only	72 (28.0)
Resection with reconstruction	
Fibular flap	63 (24.5)
PMMF	71 (27.6)
ALT	11 (4.3)
DCIA	4 (1.6)
Latissimus dorsi flap	4 (1.6)
Others	3 (1.2)
Implant utilization	
None	151 (58.8)
Yes	106 (41.2)

*PMMF: pectoralis major myocutaneous flap; *ALT: anterolateral thigh flap; *DCIA: deep circumflex iliac arteryperforator flap.

During the postsurgical period (3 months), infection was found in 60 patients with an incidence of 23.3% and was categorized into five types: 1) swelling and pyometra in 15 patients (25%), who were treated with conservative therapeutics consisting of debridement (removal of the necrotic soft tissues), daily dressing changes that continued for about two weeks, and antibiotic prescription, which achieved good outcomes; 2) soft tissue split with infection in 13 patients (21.7%), of whom 10 patients received conservative therapeutics, and the remaining three patients underwent extended resections in conjunction with flap re-coverage; 3) fistula formation with titanium plate exposure (n = 16, 26.7%) where removal of titanium plates was adequate for 12 patients, and the other four patients needed extra flap coverage; 4) fistula formation with bone exposure in seven patients (11.7%), five of whom received curettage of the necrotic bone and subsequent conservative treatment, and the remaining two patients received bone ablation and flap reconstruction; and 5) partial necrosis of the vascularized flap with infection (n = 9, 15%), for which seven patients received conservative treatment, and the remaining two patients needed new flap coverage (Table 4). All infection cases were treated with surgical intervention.

**Table 4:** Postoperative infection and corresponding treatments.

Type	No. of cases (total = 60)	Treatments
Swelling and pyometra	15 (25%)	Conservative method in 15 cases
Tissue split with infection	13 (21.7%)	Conservative method in 10 cases
		Extensive resection and FRC in 3 cases
Fistula and plate exposure	16 (26.7%)	Plate removal and wound care in 12 cases
		Plate remove and FRC in 4 cases
Fistula and bone exposure	7 (11.6%)	Debridement of necrotic bone in 5 cases
		Bone debridement and FRC in 2 cases
Flap partial necrosis and infection	9 (15.0%)	Conservative method in 7 cases
		Extensive resection and FRC in 2 cases

*Conservative method: debridement (remove of the necrotic soft tissues), dressing change everyday continued for about two weeks, and antibiotic prescription; *FRC: flap re-coverage.

### Predictors for postoperative infection in univariate and logistic analysis

A total of 257 patients were included in the predictive analysis. All predictive factors were analyzed to identify their correlation with PPI. In the univariate analysis, six factors demonstrated a significant association with PPI with p-values less than 0.05 (Table 5). Other factors failed to show a statistically significant association with PPI in the chi-square test. When these six factors were further entered into the logistic model, only four variables maintained their predictive role in multiple logistic regression analysis. The patients who received a radiation dose $\geqslant$80 Gy were 2.044 times more likely to develop PPI than those who received a dose <80 Gy (p = 0.035, 95% CI: 1.050–3.979). Patients diagnosed with bilateral ORNM were 4.120 times more likely to develop PPI than those with unilateral ORNM (p = 0.006, 95% CI: 1.501–11.307). The odds of susceptibility to PPI in patients with a skin fistula were 3.078 times the odds of cases without infection (P = 0.040, 95% CI: 1.023–9.203). The risk of PPI increased abruptly with an OR of 2.115 (P = 0.020, 95% CI: 1.125–3.976) when an implant (titanium plate) was used for fixation during the operation (Table 6).

**Table 5:** Perioperative factors associated with PPI of ORNM in univariate analysis.

Variable	Total	Infection	Non-infection	Odds ratio	95% CI	P
Radiation Dose						
<80 Gy	184 (71.6)	32	152	1 (reference)		
$\geqslant$80 Gy	73 (28.4)	28	45	2.956	1.611–5.421	<0.001
Unilateral/bilateral ORNM						
Unilateral	237 (92.2)	48	189	1 (reference)		
Bilateral	20 (7.8)	12	8	5.906	2.286–15.257	<0.001
Skin fistula						
None	145 (56.4)	24	121	1 (reference)		
Yes	112 (43.6)	36	76	2.388	1.323–4.312	0.003
Orocutaneous fistula						
None	165 (64.2)	31	134	1 (reference)		
Yes	92 (35.8)	29	63	1.990	1.105–3.583	0.021
Pyorrhea						
None	135 (52.5)	23	112	1 (reference)		
Yes	122 (47.5)	37	85	2.120	1.173–3.831	0.012
Implant utilization						
None	151 (58.8)	26	125	1 (reference)		
Yes	106 (41.2)	34	72	2.270	1.262–4.084	0.006

**Table 6:** Logistic regression analysis of significant factors detected in univariate analysis.

Viable	Exp (B)	95% CI	P value	Points contributed
Radiation Dose				
<80 Gy	1 (reference)			0
$\geqslant$80 Gy	2.044	1.050–3.979	0.035	4
Unilateral/bilateral ORNM				
Unilateral	1 (reference)			0
Bilateral	4.120	1.501–11.307	0.006	8
Skin fistula				
None	1 (reference)			0
Yes	3.078	1.050–9.023	0.040	6
Orocutaneous fistula				
None	1 (reference)			—
Yes	0.477	0.150–1.520	0.211	—
Pyorrhea				
None	1 (reference)			—
Yes	1.666	0.757–3.665	0.205	—
Implant utilization				
None	1 (reference)			0
Yes	2.115	1.125–3.976	0.020	4

### Nomogram model establishment and its performance

**Figure 5:**
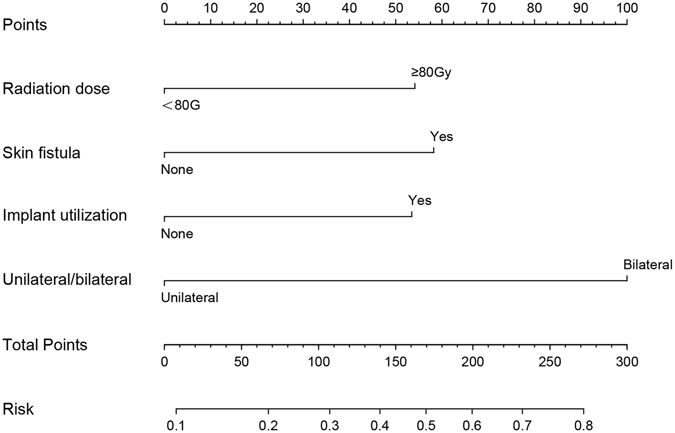
Nomogram model for predicting the risk of postoperative infection. Every patient was assigned a total point value and corresponding hazard risk can be calculated in the axis of risk.

**Figure 6:**
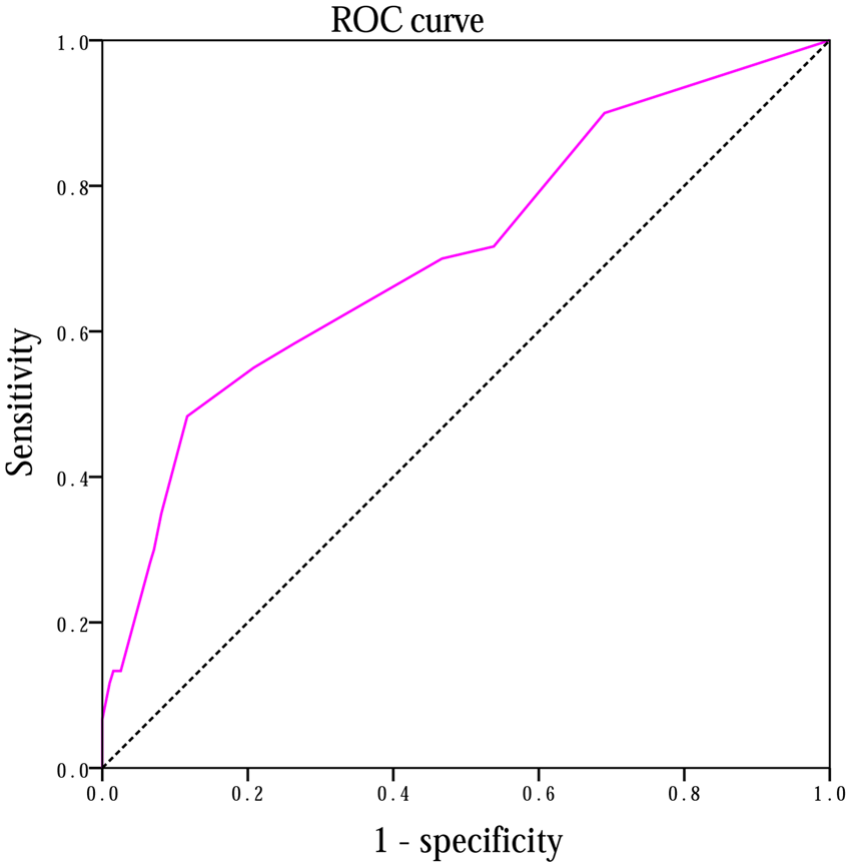
Discrimination capability of nomogram model. Receiver operating characteristic (ROC) curve showed an area under curve (AUC) of 0.708.

**Figure 7:**
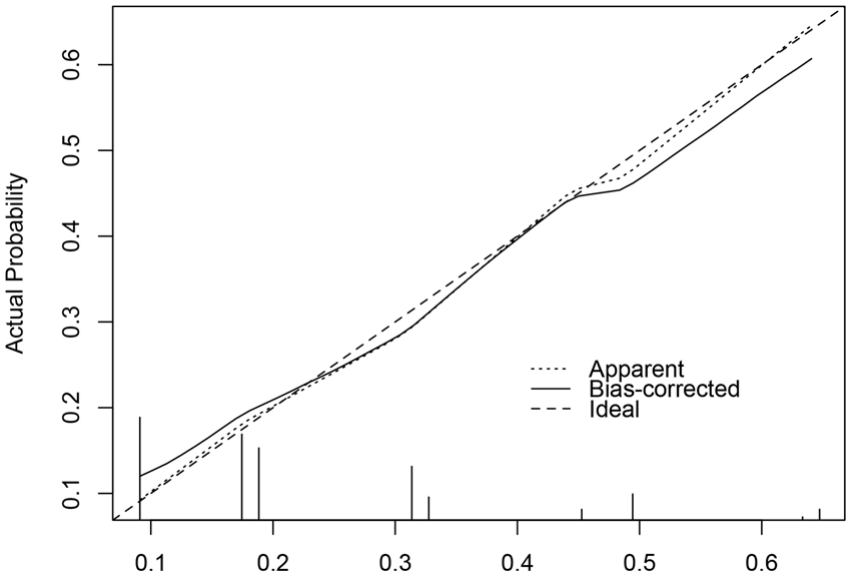
The calibration curve revealed adequate fit of the nomogram model in predicting the risk of PPI.

Based on the results obtained from multiple logistic regression analysis, a nomogram model was established to predict PPI in ORNM patients. A score was assigned to the four identified factors according to their regression coefficients (Table 6). The score sum of each predictor was calculated for each patient to form a risk-stratification score that was then separated into three categories: 1) 0–6; 2) 7–13; and 3) 14–22. We found that PPI risk increased with the stratification score. The susceptibility to PPI in patients with scores of 14–22 was 2.833 times that of patients with scores of 7–13 and 7.585 times that of patients with scores of 0–6 (Table 7). Figure 5 outlines the nomogram model in which the total points and corresponding risk of every patient were calculated in a more convenient way. We plotted the ROC curve to estimate the discriminative ability of this nomogram model depending on the AUC with an area of 0.708 (95% CI: 0.646–0.798, and the p-value of the hypothesis test of the AUC of 0.5 was <0.001), which indicated that the probability that a patient with PPI had a higher score than a patient without PPI was 70.8%, showing the power of this predictive model (Fig. 6). The calibration curve also revealed strong goodness of fit (Fig. 7).

**Table 7:** Correlation of risk-stratification score with PPI.

Group	Score	Total	Infection	No-infection	Single trend test p-value	Odds ratio	95% CI
1	0–6	170 (66.1)	25	145	—	1 (reference)	—
2	7–13	57 (22.2)	18	39	0.006 (VS group1)	2.677	1.327–5.399
3	14–22	30 (11.7)	17	13	0.021 (VS gourp2)	2.833	1.137–7.059
					<0.001 (VS group1)	7.585	3.282–17.527

*VS: versus.

## Discussion

Mandibular osteoradionecrosis is a radiation-induced late toxicity that is among the most terrible side effects affecting the quality of life of survivors of head and neck cancers. Thus far, most investigations have dealt with the modality of ORNM therapeutics and risk factors related to the causation of ORNM^1, 3–6, 9, 10^. Few studies have probed the postoperative complications of ORNM^7, 11–14^, and almost no literature has systemically identified the prognostic factors for PPI or proposed corresponding prophylaxis and intervention protocols. This imbalance in research calls for in-depth evaluation of PPI to standardize therapeutics and minimize postoperative morbidities. Our sample size of 257 patients is the largest of any ORNM investigation to date^11, 15^. Through data review and analysis, we found that four factors (radiation dose, skin fistula, unilateral/bilateral involvement, and implant utilization) were predictors for PPI of ORNM. Other factors failed to demonstrate a statistical association with PPI. Based on these findings, we established a risk-stratification score and nomogram model to predict the risk of PPI occurrence; these tools can be used to improve clinical evaluation. The nomogram model was evaluated for discrimination capability and found to have an AUC of 0.708. Previous studies have reported a PPI incidence ranging from 11.1% to 22.2% in reconstruction of ORNM with a vascularized flap^11, 14, 16, 17^. In the current study, the total incidence of PPI was 23.3%, and in patients reconstructed with a free flap, it was 21.7%, which is in accordance with other studies.

In our study, the average radiation dose was 75.35 Gy, which is comparable with the mean dose that was used in previous studies^17–19^. This high dosage may be attributed to the high proportion of patients with nasopharyngeal carcinoma (96/257, 37.4%), who require higher doses for primary control or local relapse. It was common for a patient to receive a cumulative dose higher than 100 Gy when malignancy was recurrent^4^. Another situation was when a patient had undergone prior surgery with radiotherapy at another institution but was referred to our department and diagnosed with tumor recurrence, thus a second course of irradiation was inevitable. For the aforementioned reasons, we set the cut-off for the radiation dose at 80 Gy and performed further analysis. Controversy persists among investigations as to whether the radiation dose is directly proportional to the development or severity of ORNM^18, 20–22^; nevertheless, no coherent conclusion has been drawn because the distinct grading system used in previous investigations made comparison baseless and meaningless. Mucke *et al*.^12^ failed to prove the predictive role of the radiation dose on postsurgical infection. In the current study, we demonstrated that the radiation dose was statistically associated with PPI in both univariate and multiple logistic regression analyses. Radiation damage to skin and connective tissue that is marked by fibrosis reduces vascularity and ultimately causes necrosis, as has been proven through histological analysis^23^. Radiation damages the immunological barrier of the skin and its self-renewing property, resulting in hypovascularized tissue that is susceptible to trauma or infection^24^.

A skin fistula is one of the most typical symptoms of ORNM. During the development of ORNM, necrotic bone may suffer from infection, and irradiated soft tissue may easily split, thus forming a skin fistula with infection and hindering the healing capability of surrounding tissue even after an extended bone resection is performed^7^. In the current study, the presence of a skin fistula prior to surgical treatment was a major risk factor for PPI in both univariate and multiple logistic regression analyses. However, mucosal defects failed to show the same effect, and orocutaneous fistulae demonstrated significance in the univariate analysis but not in the multiple logistic regression analysis. These results indicate a decisive effect of skin fistulae on the significance of orocutaneous fistulae in the chi-square test, but that effect was absent in multivariate analysis when all factors were considered. Skin fistulae have a worse microenvironment and lower resistance after radiation than skin with an intact barrier, and this chronically infected tissue may increase the propensity of PPI^24^. Complications from infection are usually associated with necrotic wound tissue (ulcers of the mucosa, skin fistulae) before surgery^11^.

Bilateral ORNM may result from extensive irradiation covering both sides of the mandible. In our cases, ORNM with bilateral involvement accounted for 7.8% of cases (20/257), and these patients were more prone to suffer from PPI than those with unilateral ORNM (OR = 4.12, p = 0.006, 95% CI: 1.501–11.307). This correlation was not analyzed in the study of Mucke *et al*.^12^, who attempted to find prognostic factors for PPI. Extensive irradiation imposed damage not only on bone tissue but also on the surrounding soft tissue and vascular system. The metabolism and self-refreshing capability of these irradiated soft tissues were significantly depressed, with the consequence of lower resistance to infection^24^. In the current study, little evidence was gathered regarding the predictive role of the severity of ORNM on the onset of PPI; the result was similar to the findings of Mucke *et al*.^12^. However, the staging system used in our cases was not the same, indicating that even when different clinical staging systems were evaluated, PPI had no significant correlation with the severity of ORNM. It is easier to control necrotic bone than to address the extensive destruction of irradiated soft tissue. Postoperative infection may directly correlate with soft tissue damage rather than bony destruction.

Reconstruction plates are commonly used to stabilize both sides of the remaining mandible or bind the interface between the mandible and bone graft. The disadvantage of this biomaterial is that postsurgical plate exposure increases skin fistula formation, with an incidence of 7.1% to 46.1%^14, 25^. The ratio in the current study was 15.1% (16/106), and we found that reconstruction plate utilization was a predictor of PPI in both univariate and multiple logistic regression analyses. The reasons for this result are as follows: 1) rejection of the titanium plate; 2) shrinkage of the grafted flap and local movement of the residual mandible rendering plate tortuosity^25^; 3) pressure on the overlying soft tissue caused by a plate in a wound during contraction^26^; and 4) sites where screws were fixed in bone tissue within prior radiation fields, where the metabolism of the bone was lower than that in bone that did not receive preceding radiotherapy^27^.

Based on our predictive model and the manifestations of PPI, we proposed prophylaxis protocols as follows. (*1*) *Preoperative assessment* (*risk*-*stratification score and nomogram model*) *of the hazard ratio for PPI is needed*, *thus preparing rigorous operative procedures and postoperative care*. (*2*) *Thorough excision of necrotic*/*infected soft tissues is needed*, *especially for skin or orocutaneous fistula and reconstruction defects with sufficient vascularized flap coverage to reduce the suture tension of binding interfaces between the flap and the remaining tissue*. It is more difficult to define the excision margin of soft tissue than of necrotic bone. Under these circumstances, extensive resections of irradiated soft tissue should be advocated even if a large flap will be needed to recover the defect^28^. Inadequate debridement of residual infected tissue may cause PPI and even progression of ORNM. Suturing the flap without retortion and decreased tension was crucial during the healing process because this measure can prevent the wound from splitting or dehiscing and can protect it from further plate or bone exposure^25^. (*3*) *Functional evaluation of recipient vessels is important*, *guaranteeing a high survival rate of the free flap and good conjunction between the flap and the remaining soft tissue*. Radiation damnification of vessels manifests in two ways: (a) pathological changes, including endothelium destruction, adventitial fibrosis and vasa vasorum damage that lead to tissue hypoxia and atherosclerosis; (b) physiological changes that result from surrounding tissue fibrosis and smooth muscle vasoconstriction, leading to a decrease in blood flow and an increase in blood pressure^29^. It is of pivotal importance to evaluate blood flow and vascular structure in a “frozen” neck through radiographic techniques (Computed Tomographic Angiography or Doppler) to select ideal recipient vessels and furnish an ample blood supply to ensure flap survival. (*4*) *We advocate the use of the mini*-*plate system to lessen plate*-*related complications*. In a retrospective study of 544 patients with fibular grafts combined with mini-plate fixation, Liu *et al*.^30^ demonstrated that the exposure rate was 1.5%, and the total incidence of complications was 10.3%; these are much lower rates than those obtained using a reconstruction plate. (5) *Exhaustive debridement of necrotic bone should be performed until bleeding bone is observed*. In most cases, bone necrosis was inextricably linked with infection^31^. To be specific, bone necrosis may lead to infection of surrounding tissue, and secondary infection may facilitate the progression of necrosis. For this reason, extended resections should be carried out to prevent ORNM from continuing and to minimize the risk of PPI. (*6*) *Prophylactic use of antibiotics during postoperative care is needed*. Intravenous antibiotics with broad spectra were used in all cases, similar to the study by Chen *et al*.^11^. Although no evidence exists concerning the preventive role of antibiotics on the onset of PPI of ORNM, we recommended that the prophylactic use of antibiotics should be advocated for the following reasons: (a) most cases include infected wounds; and (b) irradiated tissue holds a lower capacity for spontaneous healing and a lower resistance to destructive stimuli. In addition, maintaining oral hygiene and using mouthwash with an antibiotic ingredient should also be advocated.

Different modalities should be used to address PPI, depending on its variable severity. There was no statistical association between different therapeutics (resection with reconstruction versus resection only, pedicle flap versus free flap, bone flap versus soft flap), and PPI. Chen *et al*.^11^ hold the view that most PPI following flap reconstruction is mild and heals well through treatment with intravenous antibiotics. In the current study, conservative therapy including local debridement, dressing changes and antibiotic prescription was performed in 53.3% (32/60) of cases and achieved positive outcomes. A previous study reported a plate removal rate of 21% and unsatisfactory cosmetic outcomes^32^. In our cases, the patients with plate exposure needing surgical removal accounted for 15.1% (16/106), and most of these patients (12/16) healed spontaneously following plate removal and local wound care; this result was in accordance with the treatment of plate problems in the study by Blackwell *et al*.^26^. However, contour deformity following primary wound closure for plate removal may be far from ideal, and primary wound closures were sometimes difficult as a result of large soft tissue defects and high tension. Previous studies^8^ have suggested that vascularized flaps should be used in the closure of large oropharyngocutaneous fistulae located in the submandibular region. For these reasons, we performed free flap coverage in four cases with plate exposure. The same situation was found in three cases that were diagnosed with soft tissue splits with infection, where two patients had local bone necrosis and two patients had partial necrosis of the flap. We concluded that, in patients with mild infections, conservative treatment should be sufficient. For patients with plate rejection and bone necrosis, modalities entailing the removal of the plate and necrotic bone combined with dressing changes and intravenous antibiotics should be advocated. More seriously, vascularized flaps should be used to reconstruct large soft tissue defects following plate removal, necrotic bone debridement or partial necrosis of the flap.

The sample size of 257 in the current study is the largest in the literature concerning ORNM. This larger sample size made the statistical results more powerful and reliable. Therapeutic methods showed better coherence in single institution investigation, thus lessening the treatment bias. There were also several limitations in current study. First, it is difficult to entirely eliminate selection and document bias in a retrospective study, and unknown confounders may affect the statistical results. Second, 24 patients (including 3 patients died during hospital stay period) were excluded due to exclusion criteria, and missing data may cause bias. Third, the results from single institutions may be less generalizable than results from multicenter investigations. Finally, the nomogram model and risk-stratification score should be tested further in prospective studies. Fourth, the selection of predictors for the model in current study was based on statistical significance testing for bivariate association, and this may miss predictors that can improve the performance of multivariable model. Fifth, we used observational data as the basis for clinical recommendations for ORNM treatment, even though observational data by themselves do not provide a sufficient basis for causal inferences. Hence, readers should view these proposed recommendations on the basis of their own expert judgment.
